# LC-MS/MS Method for Serum Creatinine: Comparison with Enzymatic Method and Jaffe Method

**DOI:** 10.1371/journal.pone.0133912

**Published:** 2015-07-24

**Authors:** Meixian Ou, Yunxiao Song, Shuijun Li, Gangyi Liu, Jingying Jia, Menqi Zhang, Haichen Zhang, Chen Yu

**Affiliations:** 1 Shanghai Clinical Research Center, Chinese Academy of Sciences, Shanghai, China; 2 Central Laboratory, Shanghai Xuhui Central Hospital, Shanghai, China; 3 Department of Clinical Laboratory, Shanghai Xuhui Central Hospital, Shanghai, China; UNIFESP Federal University of São Paulo, BRAZIL

## Abstract

Accurate quantification of creatinine (Cre) is important to estimate glomerular filtration rate (GFR). Differences among various methods of Cre quantification were previously noted. This study aims to develop a liquid chromatography tandem mass spectrometry (LC-MS/MS) method for serum Cre and compare this method with clinical routine methods. LC-MS/MS analysis was performed on API 4000 triple quadrupole mass spectrometer coupled with an Agilent 1200 liquid chromatography system. After adding isotope-labeled Cre-d3 as internal standard, serum samples were prepared via a one-step protein precipitation with methanol. The LC-MS/MS method was compared with frequently used enzymatic method and Jaffe method. This developed method, with a total run time of 3 min, had a lower limit of quantification of 4.4 μmol/L, a total imprecision of 1.15%–3.84%, and an average bias of 1.06%. No significant matrix effect, carryover, and interference were observed for the LC-MS/MS method. The reference intervals of serum Cre measured by LC-MS/MS assay were 41–79 μmol/L for adult women, and 46–101 μmol/L for adult men. Using LC-MS/MS as a reference, the enzymatic method showed an average bias of -2.1% and the Jaffe method showed a substantial average bias of 11.7%. Compared with the LC-MS/MS method, significant negative bias was observed for the enzymatic and Jaffe methods in hemolytic and lipimic samples. We developed a simple, specific, and accurate LC-MS/MS method to analyze serum Cre. Discordance existed among different methods.

## Introduction

Endogenous creatinine (Cre) clearance, which is widely used to estimate the glomerular filtration rate (GFR), is a frequently employed biomarker of kidney function [1, 2]. GFR can be estimated using Cre clearance or calculated using formulae, which depend on Cre quantification in the serum. Thus, accurate quantification of Cre is essential to estimate GFR [3–6]. To date, enzymatic and Jaffe methods are frequently used in clinical practice [7]. However, marked differences exist among these methods for lack of adequate specificity. Moreover, these methods are more prone to interference from hemolysis, lipemia, bilirubin, protein, and ketones [8–11]. Interference evaluation and comparison of the proposed method with that of other methods are necessary before the new method is applied in patient care.

A number of mass spectrometry based methods that quantify Cre in serum or plasma with outstanding specificity and sensitivity have been reported [11–15]. An ideal LC-MS/MS method applied in routine use requires a simple sample preparation, fast turnaround time, interference-free analysis, and more importantly, traceability to reference methods or standard reference materials (SRM). However, lack of appropriate investigation on interference [13], or lack of comparison to SRM [11, 14] may limit the application of methods to routine analysis of serum Cre.

In this study, we aimed to develop a simple, specific, and reliable LC-MS/MS method to measure serum Cre in routine clinical laboratory. To verify the method's accuracy, we measured the SRM from National Institute of Standards and Technology (NIST) and trueness-based external quality assessment (EQA) samples using this new method. We also compared the results of the enzymatic and Jaffe methods with that of our method to record the differences.

## Materials and Methods

### Ethics statement

The study was reviewed and approved by Shanghai Xuhui Central Hospital Ethics Committee (protocol #201230) and complied with the guidelines on research involving human subjects. All samples were collected from the serum leftovers in the clinical laboratory of Shanghai Xuhui Central Hospital. All data were analyzed anonymously. The health, security, and privacy of the patients were protected. All experimental procedures were performed in accordance with the Declaration of Helsinki.

### Chemicals and solutions

Cre (99.8% purity) and Cre-d3 (98% purity) was purchased from Sigma (St. Louis, MO, USA) and Toronto Research Chemicals (Toronto, Canada), respectively. HPLC-grade acetonitrile was obtained from Dikma Technologies Inc. (Lake Forest, CA, USA). Ammonium acetate was acquired from Shanghai Anpel Scientific Instrument Co., Ltd. (Shanghai, China). Water was prepared in house with Millipore (Merck Millipore, Germany) water purifying system. All other chemicals were of analytical grade.

Cre stock solution was prepared at a concentration of 14.57 mmol/L in water. The working solutions at various concentrations (4.4, 8.8, 17.7, 44.3, 88.5, 177.0, 442.5 and 885.0 μmol/L) were prepared by appropriately diluting the stock solution with 20% methanol. Isotope-labeled Cre-d3 was used as internal standard (IS) and prepared with 20% methanol at 132 μmol/L in working solution. All of these solutions were stored at -20°C until use.

### Sample preparation and LC-MS/MS analysis

Aliquots of 50 μL of serum samples were mixed with 20 μL of IS working solution and 200 μL of methanol in Eppendorf tube and the mixture was vortexed for 30 s. After centrifugation at 15 000 rpm for 3 min, 50 μL of supernatant was mixed with 50 μL of water. Then the mixture was transferred into a sample vial. Subsequently, 3 μL of the mixture was injected into the LC-MS/MS system.

Chromatography separation was performed on an Agilent 1200 series liquid chromatography system consisting of G1312A binary pump, G1367B Hip ALS autosampler, G1379B degasser and G1316A TCC column oven modules (Santa Clara, CA, USA). A Hypersil Silica (5 μm, 2.1×100 mm, Thermo, USA) was used as the analytical column eluted with 45% 5 mmol ammonium acetate in water and 55% methanol at 0.3 mL/min. The total run time of the method was 3.0 min.

An API 4000 triple quadrupole mass spectrometer (Applied Biosystems/MDS Sciex, Toronto, Canada) equipped with electrospray ionization source was employed for the analysis. Cre was detected in positive multiple reaction monitoring mode using Q1/Q3 ion transitions at m/z 114.0/86.0 amu as quantifier and m/z 114.0/44.1 amu as qualifier. IS was detected at m/z 117.0/89.0 amu. Maximum permitted tolerance was set within ±20% for quantifier/qualifier ratio in ion intensity. The source parameters were set as follows: curtain gas, 25 arbitrary unit; CAD, 12 arbitrary unit; GS1, 65 arbitrary unit; and GS2, 40 arbitrary unit. The decluster potential, entrance potential, collision energy, collision cell exit potential were optimized as 50 V, 4 V, 17 V, and 8 V, respectively, by infusing standard Cre and Cre-d3 solutions (both at 1 μg/mL in methanol) into the mass spectrometer. Capillary voltage was set at 4.5 kV, and the source temperature was set at 550°C. Analyst 1.5 software (Applied Biosystems/MDS Sciex, Toronto, Canada) was used for equipment control, data acquisition and report processing. Cre was quantified by calibrating for each run by using a 1/x weighted least square regression.

### Method validation

Method validation included the evaluation of the matrix effect, candidate matrix selection, interference, linearity, precision, accuracy, carryover, and stability by referring to the CLSI guidelines EP10-A3 [16], EP7-A2 [17], C50-A [18] and FDA Guidance for Industry Bioanalytical Method Validation [19].

Matrix effect was evaluated using a post column infusion approach. Pure IS solution (132.7 μmol/L in 20% methanol) was infused using a syringe pump at 20 μL/min via the T-connection to the analytical column. Simultaneously, serum samples collected from three males and three females were injected by an autosampler. A decrease or increase of the baseline of Cre-d3 at the retention time indicated ion suppression or ion enhancement.

Given the endogenous nature of Cre, a “blank” serum is unavailable. Therefore, selecting a candidate matrix for the preparation of calibrators and dilution of highly concentrated samples is necessary. To verify the suitability of 20% methanol for this purpose, a pure solution of analyte was solved in 20% methanol and spiked at the upper reference limit. Samples from three males and three females were subsequently prepared. The solutions mentioned above were mixed at 1:1 (v/v) ratio. A percentage difference of Cre to IS signal ratio between the mixed samples and theoretical value (average of real serum samples and spiked analyte) within ±20% is considered suitable as candidate matrix.

The potential interference from endogenous compounds was evaluated by comparing the Cre to IS signal ratio of mixed samples with lipimic, hemolytic, icteric, and uremic samples. Cre was spiked to 20% methanol at the upper and lower ends of the reference range. Subsequently, they were mixed with endogenous interference samples at 1:1 (v/v) ratio. Three groups of samples including spiked, mixed, and endogenous interference were prepared at once. A less than 20% percentage difference in signal ratio between mixed samples and the average of spiked and endogenous interference samples was considered no significant interference. The interference process was based on the CLSI guideline EP7-A2 (Interference Testing in Clinical Chemistry).

Linearity was evaluated by analyzing samples prepared within the concentration range of 4.4 μmol/L to 885.0 μmol/L in 20% methanol. Linearity is considered acceptable if the analytical accuracy falls within 85%-115%, and when the coefficient of variation (CV) falls within ±15%. However, the linearity of the lower limit of quantification (LLOQ) is acceptable if accuracy is within 80%-120% and CV is within ±20%. Evaluating linearity was mainly based on the principle of FDA guidance (Bioanalytical Method Validation).

LLOQ is the lowest concentration that could be measured with acceptable accuracy (80%-120%), CV (±20%), and with signal to noise ratio (S/N) greater than 10. LLOQ requirements were based from the CLSI guideline C50-A (Mass Spectrometry in the Clinical Laboratory: General Principles and Guidance).

Precision was evaluated by analyzing QCs at three levels in pooled patient samples. Intra-assay precision as determined by analyzing QCs of 20 replicates within a run, whereas inter-assay precision was determined by analyzing QCs of three replicates per run, two runs per day, for 5 days. Precision was evaluated according to the CLSI Guideline EP10-A3 (Preliminary Evaluation of Quantitative Clinical Laboratory Measurement Procedures).

Accuracy was assessed by determining the SRM 1950 purchased from NIST for three replicates per run in three runs. A percentage deviation from certified concentration <15% is accepted.

A high sample at a minimum of twice the upper limit and a low sample at 20% above the lower limit of the analytical measurement range were prepared in 20% methanol. Low sample was sequentially injected before and after the high sample. The calculated difference within ±20% between the two low samples indicated no significant carryover. This process was according to the approved CLSI Guideline EP10-A3 (Preliminary Evaluation of Quantitative Clinical Laboratory Measurement Procedures).

Stability was investigated under various conditions. Bench-top stability was evaluated by placing serum samples on bench top at room temperature for 24 h. Freeze-thaw stability was evaluated using three cycles of varying temperature from -20°C to room temperature. Autosampler stability was assessed by placing processed serum samples in an autosampler at room temperature for 36 h. Long-term stability was evaluated by freezing serum samples at -20°C for 4 months.

### Method comparison

Leftovers of serum samples were collected from the clinical laboratory of Shanghai Xuhui Central Hospital for method comparison. These samples were obtained from 100 males (57.0 ± 21.1 years, range: 18–92 years) and 262 females (61.6 ± 13.9 years, range: 18–94 years). Meanwhile, leftovers of 50 hemolytic serum samples and 50 lipimic samples (triglycerides: 1.88–17.6 mmol/L) were collected. All samples were split into three aliquots and stored at -20°C until analysis.

The samples were simultaneously analyzed with LC-MS/MS, enzymatic and Jaffe methods for serum Cre. Prior to analysis, the enzymatic and Jaffe methods were calibrated with calibrators and then internal quality controls (lyophilized quality control serum at normal level and disease level, Mindray, Shenzhen, China) were measured (acceptable biases 4.3%) according to respective manual instructions and procedures. The enzymatic and Jaffe methods were performed on a BS 800 automatic biochemical analyzer (Mindray, Shenzhen, China). The detection range of the enzymatic method was 10–9000 μmol/L with endpoint reaction mode at 546 nm wavelength and reaction temperature at 37°C. The detection range of the Jaffe method was 9–2420 μmol/L with fixed time reaction mode at 510 nm wavelength and similar reaction temperature.

### Statistics

Deming regression, Pearson’s correlation and Bland-Altman plot were employed by Analyse-it software for Excel (version 2.30, Analyse-it Software, Ltd). Difference were compared using ANOVA by SPSS software (version 22.0, IBM). *P*<0.05 is considered as statistically significance.

## Results

### Method validation

**Fig 1:**
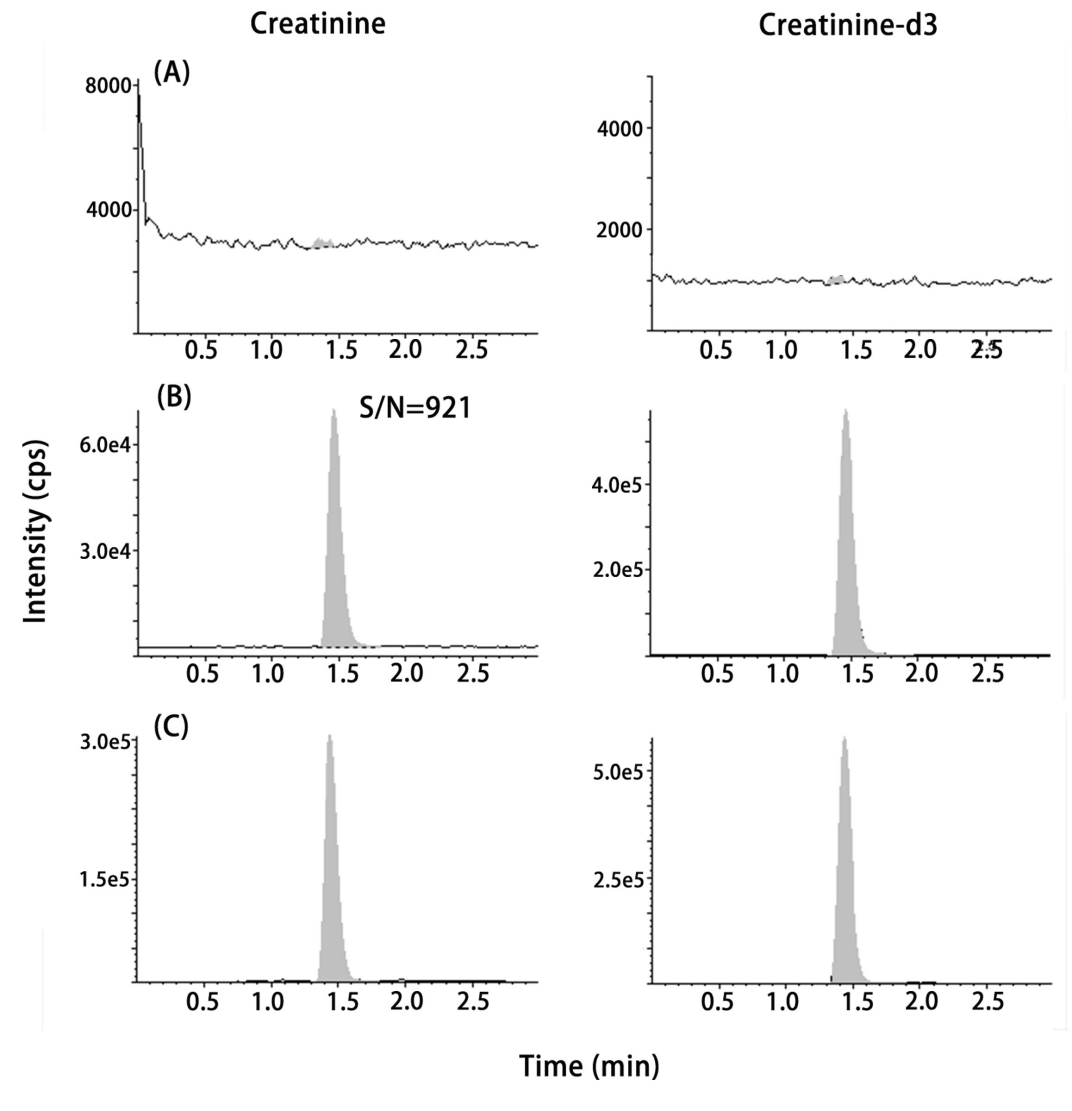
Chromatograms of (A) blank (20% methanol), (B) LLOQ (4.4 μmol/L, S/N = 921) and (C) a patient sample (43.9 μmol/L).

**Fig 2:**
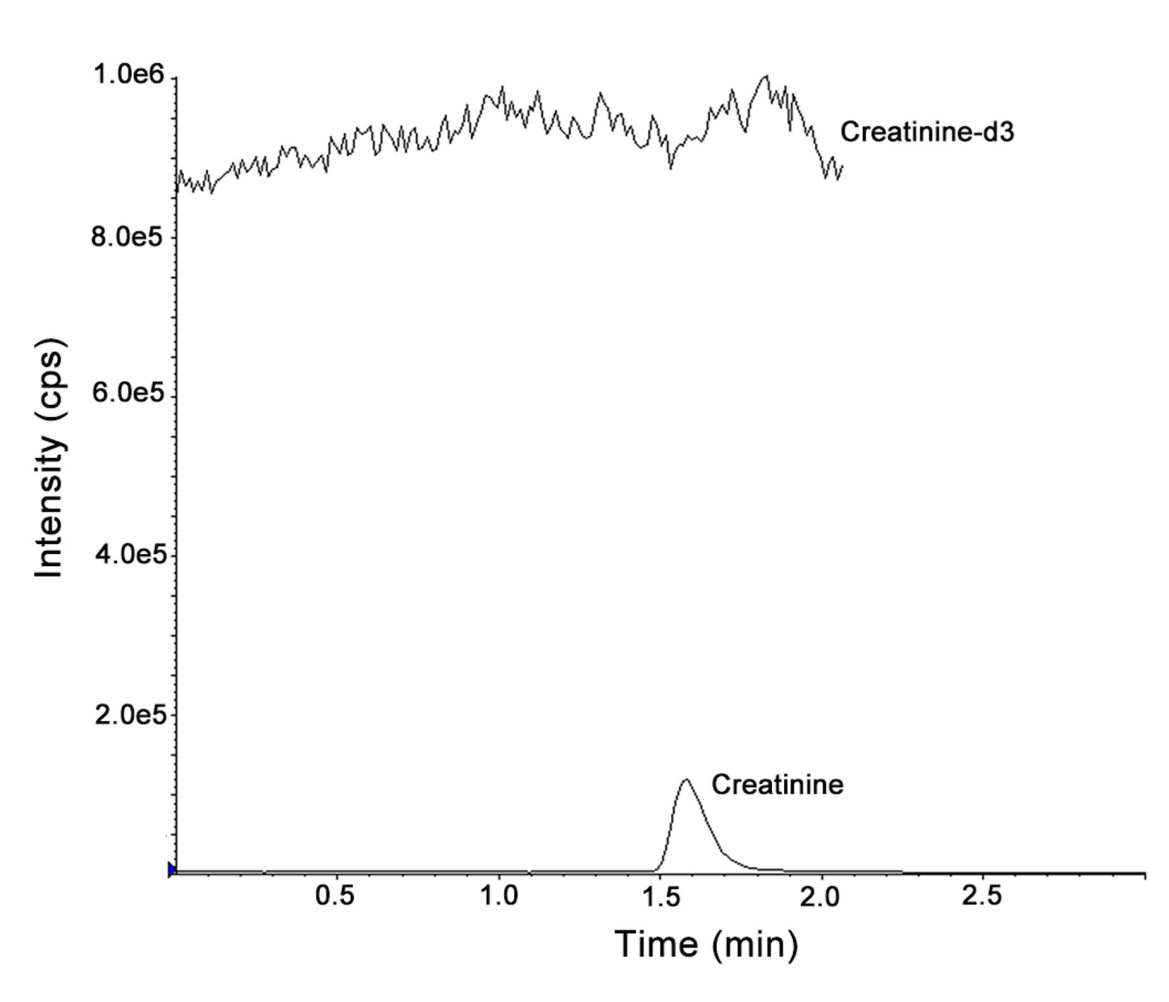
Chromatograms of post-column infusion of Cre-d3 (132.7 μmol/L in 20% methanol) with a patient sample.

Silica column eluting with applied chromatographic conditions showed that the retention time of Cre and IS was about 1.5 min within the 3.0 min liquid chromatography run time, allowing for a fast turnaround time for serum Cre analysis in routine clinical laboratory. Fig 1 shows the representative chromatograms of blank (20% methanol), LLOQ (4.4 μmol/L spiked to 20% methanol), and a patient sample (43.9 μmol/L). Fig 2 shows the chromatograms of post-column infusion Cre-d3 (132.7 μmol/L in 20% methanol) with syringe pump at 20 μL/min via T-connection to the analytical column with a patient sample. Flat baseline of Cre-d3 indicated no endogenous interference peak at the retention time of Cre and IS in patient sample, suggesting a good selectivity of this method. Moreover, no significant matrix effect and carryover were detected.

Assay result was linear, which ranged from 4.4 μmol/L to 885.0 μmol/L with an accuracy of 86.3%-104.9% and CV of 0.7%-4.6%. The LLOQ was 4.4 μmol/L with an S/N of 921 (panel B in Fig 1). The bias from the NIST SRM 1950 was 1.06% (95% CI: -1.26% to 2.45%). Moreover, we measured the trueness-based EQA proficiency test of the samples (n = 10) provided by the National Center for Clinical Laboratory of China. The bias was 1.89% (95% CI: -1.34% to 3.22%). The intra- and inter-assay precision were <3.8% (Table 1). In the mixing study by using 20% methanol as the blank matrix, the percentage bias from the theoretical value was -2.0% to 3.5%, demonstrating good interchangeability between the candidate matrix and the real serum. No significant interference was observed in lipimic, hemolytic, icteric and uremic serum samples (S1 Fig). The percentage bias was -4.95% to 9.92% between the calculated 50/50 mixture peak area ratio and actual 50/50 mixture peak area ratio.

**Table 1:** Precision of serum Cre by LC-MS/MS analysis (low level was the pool of patient samples, middle level was 35.4 μmol/L Cre spiked in low level, and high level was 132.7 μmol/L Cre spiked in low level).

	Intra-assay precision (n = 20)			Inter-assay precision (n = 30)		
Level	low	middle	high	low	middle	high
Mean (μmol/L)	75.0	110.2	201.7	75.6	109.6	201.0
SD (μmol/L)	1.2	2.1	3.1	0.9	1.5	7.7
CV (%)	1.6	1.9	1.5	1.2	1.4	3.8

Table 2 shows that Cre remained stable on the bench-top for 8 h, with three freeze-thaw cycles, in an autosampler for 24 h, and when frozen for 4 months.

**Table 2:** Stability of Cre under various conditions. Values are expressed as the bias from the initial concentration.

Creatinine concentration (μmol/L)	75.3	109.9	201.3
Bench-top for 8 h	9.2	4.1	8.4
Freeze-thaw 3 cycles	-2.8	-3.1	-0.5
Autosampler for 24 h	-0.1	-1.7	1.9
Frozen (-20°C) for 4 months	6.7	1.5	-5.9

### Reference intervals and method comparison

Serum Cre was determined using the validated LC-MS/MS method, and the reference intervals were constructed based on 95% (2.5%-97.5%) CI by using a non-parametric analysis. The reference intervals of serum Cre were 46 μmol/L to 101 μmol/L for adult men (n = 100), and 41 μmol/L to 79 μmol/L for adult women (n = 262).

**Fig 3:**
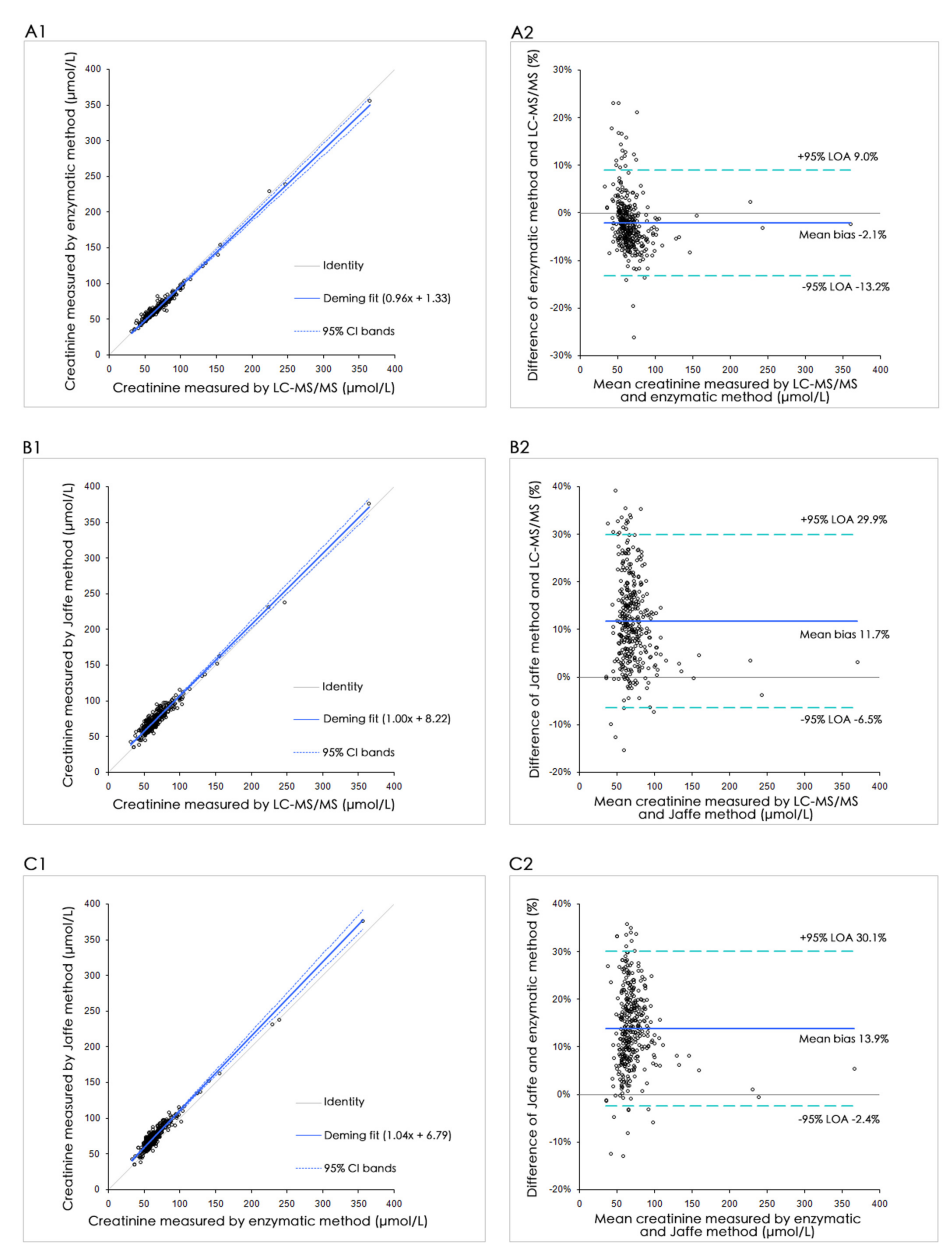
Method comparison of the serum Cre with (1) Deming regression and (2) Bland-Altman analysis of (A) LC-MS/MS versus enzymatic method, (B) LC-MS/MS versus Jaffe method, and (C) enzymatic method versus Jaffe method.

Patient serum samples collected from 100 men and 262 women were split into three aliquots, and were stored at -20^°^C until analysis. Serum Cre was measured in a single run using the LC-MS/MS, enzymatic and Jaffe methods. Results were compared by Deming regression and Bland-Altman plot (Fig 3). Enzymatic and Jaffe methods showed a mean proportional difference of -2.1% and 11.7%, respectively, compared with LC-MS/MS. In the Bland-Altman analysis, the 95% limit of agreement ranged from -13.2% to 9.0% for the enzymatic method and from -6.5% to 29.9% for the Jaffe method between the proposed LC-MS/MS assay. Between-method parameters of Bland-Altman mean differences, Deming regression analysis and Pearson's correlation coefficients are presented in S1 Table.

**Fig 4:**
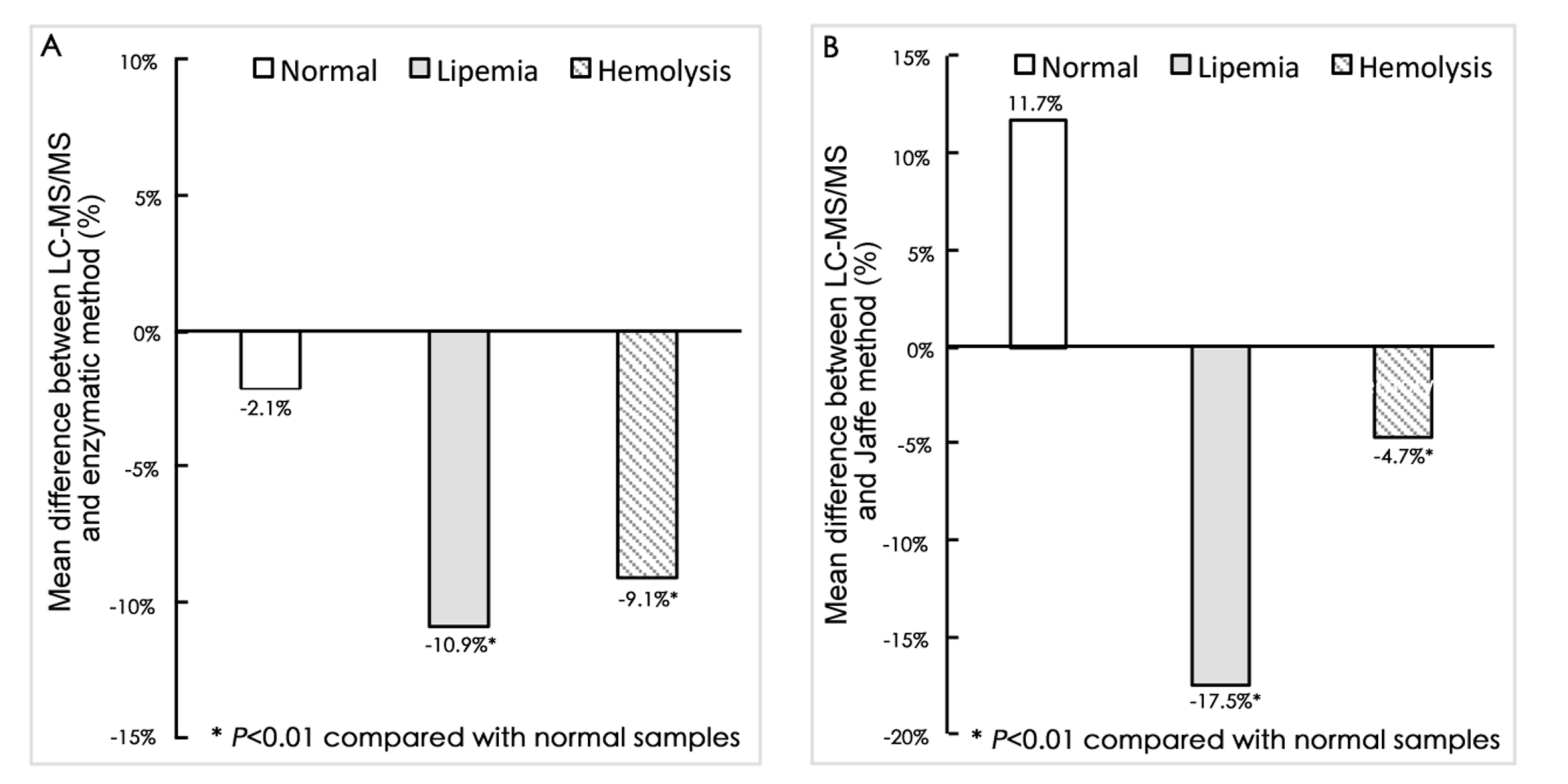
Effects of lipemia and hemolysis on the mean difference between (A) LC-MS/MS and enzymatic methods, and (B) LC-MS/MS and Jaffe methods.

We investigated the effects of lipemia and hemolysis on the results of serum Cre analysis. The serum Cre in 50 leftover hemolytic serum samples and 50 lipimic samples (triglycerides: 1.88–17.6 mmol/L) were analyzed by LC-MS/MS, enzymatic and Jaffe methods. Compared with the normal samples, a significant negative impact on the Cre results of enzymatic and Jaffe methods (Fig 4).

## Discussion

We developed a LC-MS/MS method for the quantification of serum Cre in routine clinical application, and an isotope-labeled Cre-d3 was used as IS. This method is simple, fast, accurate, and specific for Cre. Sample preparation requires a one-step protein precipitation using a 50 μL sample. We performed chromatographic separation on a Hypersil Silica column, which is slightly different to other published methods performed on reverse phase [12, 15] or ion exchange chromatography [13, 20]. The retention time of Cre and IS was about 1.5 min within the 3.0 min liquid chromatography run time, thus allowing a high throughput method alternative to routine clinical assay for Cre. As creatinine-free matrix is generally unavailable, we investigated the feasibility to use alternative matrices for the preparation of calibrators. We found 20% methanol is an ideal candidate matrix as it is simple, inexpensive, and more importantly, interchangeable to serum. It was reported by Koster et al. that three different strategies, including calculation the intercept of the calibration, using extremely high concentration calibration curve, and using calibration curve prepared with isotope-labeled standard, may also be effective solution to the problem of natural presence of Cre in real samples [21].

Results of the NIST SRM 1950 and trueness-based EQA verified the accuracy of our LC-MS/MS method with average bias of 1.06% and 1.89% respectively. This results indicates an excellent comparability to traceable materials. Cre in NIST SRM 1950 was verified using an isotope-dilution liquid chromatography mass spectrometry method approved by the Joint Committee for Traceability in Laboratory Medicine (JCTLM) as a higher-order reference measurement procedure [12]. Therefore the NIST SRM 1950 reference value has the highest confidence in its accuracy in that all known or suspected sources of bias have been investigated. But we should also note that it is not a weighed-in concentration, and is only provided with associated uncertainties that may be insufficient statistical agreement among various analytical methods. Even the NIST SRM 1950 is plasma-based material, the plasma and serum can be interchangeable by our method (bias 0.83%, n = 9). Furthermore, we investigated the matrix effect using the post-column infusion approach and the interference from lipimic, hemolytic, icteric, and uremic samples. By using Cre-d3 as the indicator, we can see from Fig 2. The baseline was flat, indicating no significant ion suppression or enhancement at the retention time of Cre in patient sample.

A number of assays are currently employed in routine laboratory analyses that measure Cre. Chemical reaction based Jaffe method and enzyme creatininase based enzymatic method are the most frequently used measurement procedures in clinical laboratories. Jaffe method measuring Cre is primarily based on the reaction with alkaline picrate. The Jaffe reaction is not very specific for Cre and many compounds, including protein, bilirubin, glucose, ascorbic acid, ketone, and pyruvate, produce Jaffelike chromogen and hence may interfere the Cre measurement results [22–24]. The enzymatic method measures Cre based on the enzyme creatininase catalyzes the conversion of Cre to creatine. Similarly, care should be taken to watch the potential interference caused by ascorbic acid, bilirubin, hemoglobin, and creatine [10, 25]. Different methods for Cre measurement demonstrate varying degrees of accuracy and imprecision. Therefore whether results obtained from these methods are comparable or whether laboratories employ similar method remain unknown; results of Cre assays were reported to demonstrate considerable variation depending on the reaction mechanism (whether Jaffe or enzymatic reaction) and manufacturer [10, 11, 26]. Recently, Fernandez-Fernandez et al. investigated the potential interconversions by using a LC-MS/MS and a GC-MS method. They observed a small but systematic conversion of creatine to creatinine during the sample preparation process and found in most cases, this interconversion reaction had no influence in the final Cre results, since the creatine concentration was very small. Only when creatine has similar or even higher level than Cre, then significant bias may occur [27].

Clearly, to produce accurate and interchangeable test results, method standardization or harmonization is crucial to accurately estimate GFR or to evaluate kidney function [28]. In general, access to a reference method or SRM of routine laboratories is limited [13]. Thus, method comparison to a highly specific and accurate method is important. Enzymatic and Jaffe methods are common automated methods used to measure serum Cre. The serum Cre results of these methods were reported to considerably vary [10, 29, 30]. Jaffe and enzymatic methods are indirect and highly dependent on the reaction mode as well as susceptible to interference by endogenous or exogenous substances [11, 31].

Method comparison results demonstrated that the enzymatic and Jaffe methods had good correlation (r = 0.990 and 0.971) to our LC-MS/MS method. However, these methods showed bias, with an average of -2.1% for enzymatic method and 11.7% for Jaffe method, from our method (Fig 3). Of the 362 samples analyzed for Cre results, 64/362 (17.7%) for Jaffe method and 4/362 (1.1%) for enzymatic method exhibited a bias beyond 20%. We further investigated the impact of lipemia and hemolysis on the mean difference among LC-MS/MS, enzymatic, and Jaffe methods. Fig 4 shows that, both lipemia and hemolysis strongly negatively affected the Cre results. The Jaffe method was more susceptible to interferences than the enzymatic method. Therefore, hyperlipidemia or hemolytic samples should avoid being measured by Jaffe or enzymatic methods. LC-MS/MS method may be a better alternative to such samples. If possible, suspicious data should be verified by the LC-MS/MS method.

## Conclusion

We developed a simple, accurate, and specific LC-MS/MS method for serum Cre. The enzymatic method more comparable to the LC-MS/MS method than the Jaffe method. Lipemia and hemolysis exhibited a strong negative effect on Cre results measured by the enzymatic and Jaffe methods. Hyperlipidemia or hemolysis samples recommend being measured by LC-MS/MS method and avoid being measured by Jaffe or enzymatic methods due to discordance between different methods.

## Supporting Information

S1 Fig Chromatograms of (A) lipimic sample, (B) hemolytic sample, (C) icteric sample and (D) uremic sample. (TIF)

S1 Table Parameters between LC-MS/MS, enzymatic and Jaffe methods (Patient serum samples, n = 362). (DOCX)
