# Supplementary figures and images for: LC-MS/MS Method for Serum Creatinine: Comparison with Enzymatic Method and Jaffe Method
Source: PLoS One. 2015 Jul 24;10(7):e0133912. doi: 10.1371/journal.pone.0133912 (PMC4514740; doi:10.1371/journal.pone.0133912)

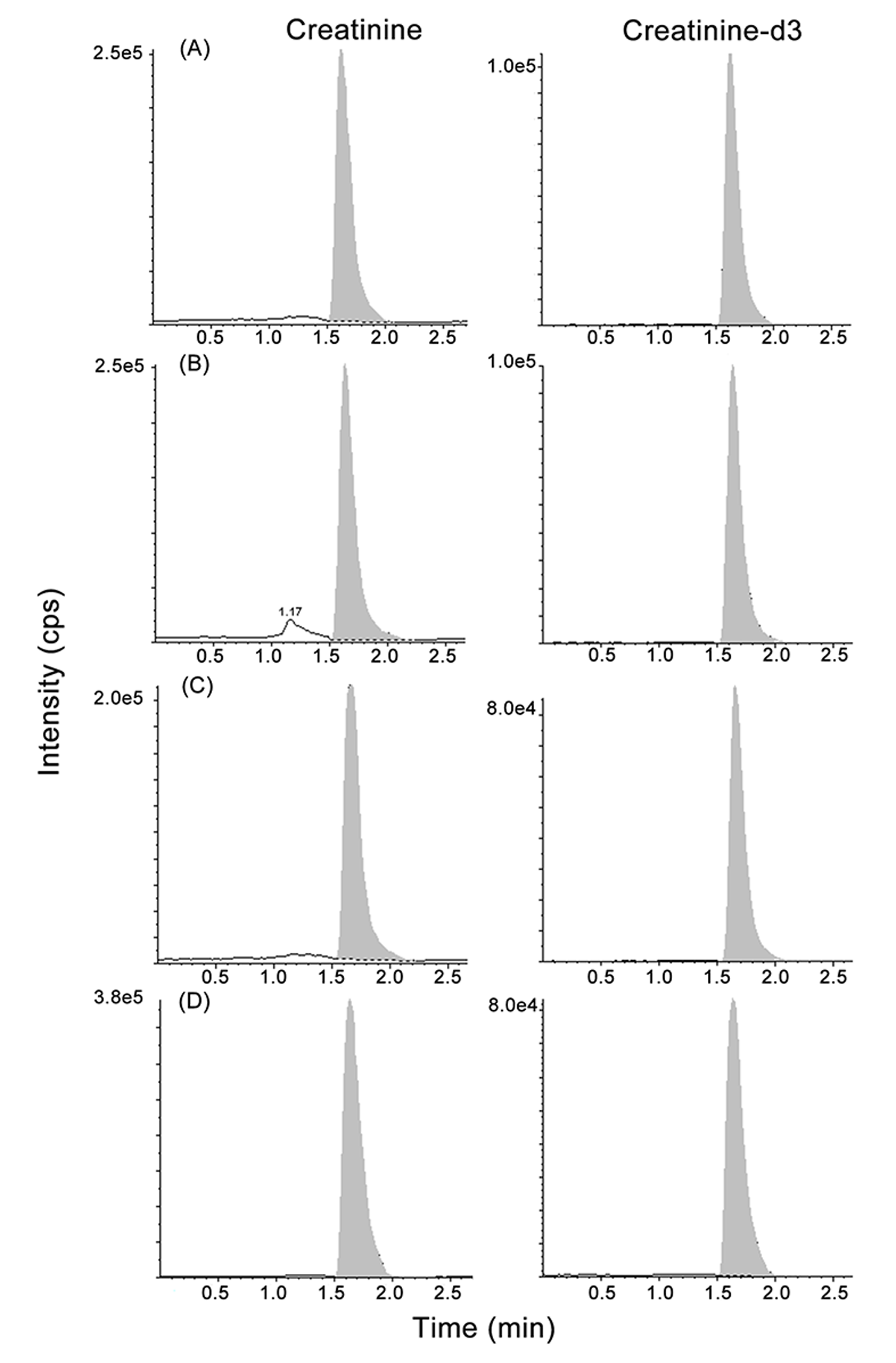

Supplement: S1 Fig — (TIF) [file pone.0133912.s001.tif]
