# Supplementary material for: LC-MS/MS Method for Serum Creatinine: Comparison with Enzymatic Method and Jaffe Method
Source: PLoS One. 2015 Jul 24;10(7):e0133912. doi: 10.1371/journal.pone.0133912 (PMC4514740; doi:10.1371/journal.pone.0133912)
Supplement: S1 Table — (DOCX) [file pone.0133912.s002.docx]

**S1 Table. Parameters between LC-MS/MS, enzymatic and Jaffe methods** **(Patient serum samples, n=362).**

| Parameters | LC–MS/MS versus enzymatic method | LC–MS/MS versus Jaffe method | Enzymatic versus Jaffe method |
| --- | --- | --- | --- |
| Constant (95%CI) | 1.33(-0.96-3.61) | 8.22(5.70-10.74) | 6.79(3.91-9.66) |
| Proportional (95%CI) | 0.96 (0.92-0.99) | 1.00 (0.96-1.03) | 1.04 (1.00-1.09) |
| Sy\|x | 3.50 | 6.17 | 5.84 |
| Bias | -2.10% | 11.70% | 13.90% |
| 95% Limit of Agreement | From -13.2% to 9.0% | From -6.5% to 29.9% | From -2.40 to 30.10% |
| Pearson’s correlation (r, 95% CI) | 0.990 (0.987-0.992) | 0.971 (0.964-0.976) | 0.974 (0.968-0.979) |
